# The Microbiome, Malignant Fungating Wounds, and Palliative Care

**DOI:** 10.3389/fcimb.2019.00373

**Published:** 2019-11-01

**Authors:** Mridula Vardhan, Zia Flaminio, Sakshi Sapru, Charles P. Tilley, Mei R. Fu, Christopher Comfort, Xin Li, Deepak Saxena

**Affiliations:** ^1^Department of Basic Science, New York University College of Dentistry, New York, NY, United States; ^2^NYU Rory Meyers College of Nursing, New York, NY, United States; ^3^Calvary Hospital, Brooklyn, NY, United States; ^4^Department of Surgery, New York University School of Medicine, New York, NY, United States

**Keywords:** cancer, malignant fungating wound, microbiome, skin microbiome, metabolomics, palliative care, pain

## Abstract

Malignant fungating wounds present in 5–14% of advanced cancer patients in the United States and are a result of cancerous cells infiltrating and proliferating in the skin. Presentation of malignant fungating wounds often occurs in the last 6 months of life and therefore become symbols of impending death for patients and their families. Due to the incurable and severe nature of these wounds, patients require palliative care until death to minimize pain and suffering. Symptoms associated with these chronic wounds include malodor, pain, bleeding, necrosis, large amounts of exudate, increased microbial growth, and more. Limited research using culture-based techniques has been conducted on malignant fungating wounds and therefore no optimal approach to treating these wounds has been established. Despite limited data, associations between the cutaneous microbiome of these wounds and severity of symptoms have been made. The presence of at least one strain of obligate anaerobic bacteria is linked with severe odor and exudate. A concentration of over 10^5^/g bacteria is linked with increased pain and exudate. Bacterial metabolites such as DMTS and putrescine are linked with components of malignant fungating wound odor and degradation of periwound skin. The few but significant associations made between the malignant fungating wound microbiome and severity of symptoms indicate that further study on this topic using 16S rRNA gene sequencing may reveal potential therapeutic targets within the microbiome to significantly improve current methods of treatment used in the palliative care approach.

## Introduction

Palliative care becomes the primary focus when treating advanced cancer patients; most of the effort and attention of the care team focuses on alleviating pain, treating symptoms, and maximizing comfort (Merz et al., 2011). Of the advanced cancer patients in the United States, 5–14% are affected by malignant fungating wounds (Dowsett, 2002; Naylor, 2002; Krathen et al., 2003; Alexander, 2009a; Grocott et al., 2013). Malignant fungating wounds (MFWs) are a result of cancerous cells infiltrating epithelium tissue and proliferating (Lund-Nielsen et al., 2011; Fromantin et al., 2013, 2014). These chronic wounds are accompanied by a combination of the following symptoms: increased microbial growth, odor, pain, exudate, impaired mobility, hemorrhage, and necrosis (Grocott, 2000; Lund-Nielsen et al., 2011; Fromantin et al., 2014). The microenvironment in MFWs is an ideal breeding ground for various microbes due to necrosis and lack of vascularization (Lund-Nielsen et al., 2011). Despite limited data, the cutaneous microbiome of wounds has been associated with the severity or presentation of symptoms (Shirasu et al., 2009; Fromantin et al., 2013, 2014). MFWs cause great distress in patients who already face severe health concerns from advanced cancer. The odor, low mobility, exudate, and other progressive symptoms often associated with MFWs make the lives of cancer patients increasingly difficult and can be major sources of emotional distress and poor quality of life (Merz et al., 2011; Fromantin et al., 2013).

Due to the severity and debilitating character of MFWs, presentation of these wounds requires comprehensive palliative care until death. According to the World Health Organization, palliative care is an “approach that improves the quality of life of patients and their families facing the problem associated with life-threatening illness, through the prevention and relief of suffering by means of early identification and impeccable assessment and treatment of pain and other problems, physical, psychosocial and spiritual.” (World Health Organization, 2019) Palliative care of MFWs focuses on alleviating the most distressing symptoms, including malodor, pain, and exudate (Shirasu et al., 2009; Fromantin et al., 2013, 2014). Generally, MFWs do not heal (Lund-Nielsen et al., 2011), and there is no standard method of treatment proven to effectively treat symptoms (Tamai et al., 2013) and dress these wounds (Fromantin et al., 2014). Existing literature reveals associations made between microbial growth in the wounds and various symptoms (Shirasu et al., 2009; Fromantin et al., 2013, 2014), but explores this relationship using culture-based techniques which yield biased results (Misic et al., 2014). The purpose of this review is to bring light to this understudied subject, investigate the available evidence regarding the relationship between microbiota and MFWs, and highlight the importance of using 16S rRNA gene sequencing to fill the gap in our understanding of the microbiome's effects on wound healing. This initial step will allow for significant advancements to be made in the effectiveness of palliative treatment of MFWs.

## Cancer and MFWs

### Afflicted Population

MFWs can present in one of three different cases: primary skin neoplasms, local extension and integumentary erosion from primary tumors or malignancy recurrence, or metastatic cutaneous lesions (Cormio et al., 2003; Seaman, 2006; Alexander, 2009a). Although any underlying malignancy can lead to MFWs, they present most often in advanced, metastatic cancers, particularly in breast cancer patients (Thomas, 1992). MFWs of the breast are the most commonly observed (66%) and are followed in prevalence by the head and neck (24%), groin, genitals, and back (3%), and other sites make up the last 8% (Thomas, 1992).

### MFW Symptoms

MFWs result from the proliferation of malignant cells that infiltrate the skin, blood, and lymph vessels in patients with advanced cancer (Naylor, 2002; Alexander, 2009a; Grocott et al., 2013). MFWs are persistent, visual markers of underlying neoplastic disease (Thomas, 1992; Cormio et al., 2003). These wounds predominantly develop during the last 6 months of life and become symbols of impending death for the patients and their families (Thomas, 1992; Merz et al., 2011). MFWs have several distressing symptoms: pain, malodor, exudate, bleeding, pruritus, perceived wound status, perceived bulk effect, and lymphedema (Alexander, 2009a; Fromantin et al., 2013, 2014; Tamai et al., 2013). These wounds and their symptoms have a detrimental effect on several aspects of the patient's life in their last few months (Grocott et al., 2013). The malodor associated with MFWs has a significant negative effect on QOL and often inflicts a sense of shame due to the pervasive and pungent smell (Merz et al., 2011). Large volumes of exudate drastically affect the management of the wound and can also contribute to this sense of shame associated with the wound (Merz et al., 2011). The excess exudate can cause further degradation of the peri-wound skin (Tamai et al., 2016). The bleeding from MFWs can be spontaneous or provoked by events such as dressing changes (Merz et al., 2011). The constant possibility of a spontaneous fatal hemorrhage due to extreme swelling has been described as “living with a time bomb” (Julia, 1998). The proliferation of cells in the wound can lead to compression or eroding of nerves and result in somatic or neuropathic pain (Naylor, 2001). The physical, psychological, and functional health of patients afflicted with MFWs is heavily affected; thereby depriving patients of a “good death” (Julia, 1998; Costello, 2006; Alexander, 2009b). Despite limited data, symptoms such as pain, odor, and exudate, as well as the severity of these symptoms, have been associated with the microbiome (Shirasu et al., 2009; Fromantin et al., 2013, 2014) making the expansion of our understanding of the relationship between the microbes in these wounds and the resulting symptoms of great significance.

### Presentation in Comparison to Other Chronic Wounds

MFWs are relatively rare (Fromantin et al., 2014), making their study challenging. Despite this, some studies have identified differences between MFWs and “regular” chronic wounds, often in terms of polymicrobial growth. A 2011 study claims that cancer's progressive nature continuously influences MFWs and results in increased amounts of tissue death and exudate (Lund-Nielsen et al., 2011). This environment becomes an ideal breeding ground for wound pathogens, making continuous colonization of MFWs an expectation. This study found that the use of honey and silver dressings, which normally have antimicrobial effects on other chronic wounds, do not influence the qualitative bacteriology of MFWs. This finding supports the idea that wound pathogens continuously colonize MFWs, rendering the effects of antibiotic and antimicrobial substances null.

Limited comparable data exists to examine the differences in the microbiome of MFWs and other chronic wounds because of the differences in techniques (culture-based vs. culture-independent). One study claims to have detected a difference in biofilm presentation between MFWs and chronic wounds. A 2013 study by Fromantin observed biofilm in 35% of their MFW samples (Fromantin et al., 2013) and cited a study which found that 60% of various chronic wounds are affected by biofilm (James et al., 2008). The Fromantin study does not explain this difference, but biofilms have historically been hard to detect without biopsy, so the detection may have been an underestimation. As discussed further on in this review, biofilm has been associated with delayed healing (Schierle et al., 2009), so a difference in biofilm presentation may be noteworthy. It is important to note that both the James and Fromantin studies were relatively small and used culture-based techniques to examine the microbiome of MFWs, which do not yield comprehensive results regarding the cutaneous microbiome (Misic et al., 2014). Conclusions based on these studies should be carefully examined and further data should be collected using culture-independent techniques.

### Current Treatment Limitations

Palliative care for MFWs is under investigated; there is no proven treatment to maximize symptom alleviation due to the wide range of symptoms and severity (Tamai et al., 2013). The heterogeneous nature of MFWs often requires treatment to be determined on a case-by-case basis (Merz et al., 2011). Dressing comprises the majority of the palliative care efforts for these wounds and requires a material that is thin (to optimize mobility), absorbent (to have the capacity to manage large amounts of discharge), has the ability to evaporate fluids, and address the issues of odor and microbial growth (Merz et al., 2011). No dressing has been developed to fulfill all these needs as well as the developing nature of the wound (Fromantin et al., 2014). Dressing changes are most often painful and uncomfortable for patients; dressings should be left on for as long of an interval as possible (Merz et al., 2011). Debriding the wound is another avenue of treatment but must be done with caution as debridement might cause unnecessary pain for a patient who has limited life expectancy (Merz et al., 2011).

Several studies have investigated the efficacy of using topical antimicrobials like metronidazole for odor management in MFWs due to their efficacy in killing anaerobes. A study in 2018 compared the efficacy of metronidazole and polyhexamethyl biguanide (PMBG) and found no significant difference in the reduction of odor an QOL of the 24 MFW patients who completed the study, but that the use of either antimicrobial achieved odor control by day four in 83.33% of patients (Villela-Castro et al., 2018). This study notes that PMBG may be the better option for malodor treatment since its cheaper and is not vulnerable to bacterial resistance like metronidazole. Another study in 2016 also found a high rate of odor control with the use of metronidazole: 95.2% of patients experienced relief in the severity of malodor (Watanabe et al., 2016). The positive results of these studies further support the inquiry regarding the microbiome's influence on the severity of MFWs. Limitations of both studies include lack of a standardized tool to measure odor which could lead to biased results. Additionally, neither study addressed the issue of biofilm, which may impede the efficacy of topical antimicrobial treatments.

The severe and perceivable symptoms can result in patients withdrawing from family and isolating themselves (Merz et al., 2011). Thus, a crucial component of the care for these wounds is integrating family members in the treatment; this will help prevent isolation and give both the family and the patient reassurance that the situation can be improved (Merz et al., 2011).

## Microbiome and MFWs

### Role of Bacteria

**Figure 1:**
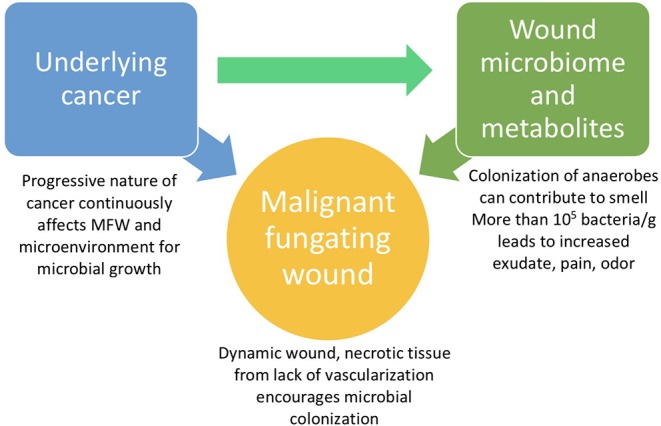
Simplified relationship between the main contributors to MFW symptoms.

The microbiome appears to be a significant factor in the severity of MFWs and their assessment for effective treatment, despite limited detailed knowledge regarding how the microbiome is involved with these wounds (Figure 1). Currently, the microbiota of MFWs has been studied using culture-based methods, which often yields results that underestimate the complexity of polymicrobial communities akin to that of MFWs (Misic et al., 2014). However, even with this incomplete data collection method, the Fromantin study in 2013 revealed that the strains of microbes, quantity of microbes, and diversity of the microbiome seem to affect the severity of the wounds. This study showed that the presence of at least one strain of obligate anaerobic bacteria, but not any strain in particular, has been linked with severe odor (*p* = 0.009) and exudates (*p* = 0.05) (Fromantin et al., 2013). Fromantin et al. also showed that there is a relationship between the concentration of microbes and symptoms. Wounds having a bacterial concentration over 10^4^/g are associated with odor (*p* = 0.02) and having over 10^5^/g bacteria is a significant threshold for an increased level of pain (*p* = 0.04) and exudates (*p* = 0.07) (Fromantin et al., 2013). This study observed that having more than four distinct species of bacteria in the wound microbiome increases the risk of odors (*p* = 0.0008) and exudates (*p* = 0.007) significantly: from 43.5 to 84.2% and from 56.5 to 86.8%, respectively (Fromantin et al., 2013). Due to the significant connection between severity of symptoms and bacterial concentration in MFWs, a potential therapeutic route could be to reduce the microbial concentration.

Biofilms are another fairly common attribute of MFWs, with one study reporting that 35% of the wounds presented with biofilm (Fromantin et al., 2013). This study did not find a correlation between the presence of a biofilm and any specific strain of bacteria or the total quantity of microbes; instead, the investigators hypothesize that biofilm is a consequence of a diverse wound microbiome. The same study observed that none of the four wounds that exhibited re-epithelialization had a biofilm, which may imply that biofilm presence slows the healing process of MFWs (Fromantin et al., 2013). The only symptom that seemed to be affected by the presence of a biofilm was a decrease in the risk of provoked hemorrhage.

### Association Between the Microbiome and Wound Healing in Comparison to Other Wounds

The relationship between the wound microbiome and healing is better understood in common chronic wounds, like diabetic foot ulcers (DFU), and in acute wounds than in MFWs. The following are results from several relevant studies that examine the microbiome in various cutaneous wounds.

A 2017 study used 16S rRNA gene sequencing to categorize the microbiome over time of DFUs in 100 patients (Loesche et al., 2017). This study found that the most abundant genera in DFUs, in order, are: *Staphylococcus, Streptococcus, Corynebacterium*, and *Anaerococcus*. Inter-visit weighted UniFrac distance was used to track stability. It was observed that wounds slowly become more stable and the wounds that were the most dynamic upon presentation healed faster. The researchers interpreted from the results that there is no standard for DFU microbial community and colonizing microbes are opportunistic. Consequently, instability of the wound microbiota is a sign of the host's effective immune defense not allowing microbes settle and establish a community. If the wound microbiome is stable, the researchers in this study would conclude that the host's immune system has been overridden by the microbes, further delaying healing in DFUs (Loesche et al., 2017).

A 2016 study used 16S rDNA pyrosequencing on samples from almost 3,000 patients to examine the microbiome of four different chronic wounds: DFUS, decubitus ulcers, venous leg ulcers, and non-healing surgical wounds (Wolcott et al., 2016). The microbiomes of all four types of chronic wounds studied were found to have similar genera of bacteria: *Staphylococcus, Pseudomonas, Corynebacterium, Streptococcus* (in order of descending relative abundance). *Pseudomonas* was found to make up the highest proportion of the biofilm communities is was present in, as well as the most common microbe to form “single species” biofilm. This study cites two others that show that *Pseudomonas* is resistant to host immune responses like macrophages (Leid et al., 2005) and antibodies (Lam et al., 1987); this resistance may be what allows this bacterium to form biofilms.

A 2014 study used 16S rRNA gene sequencing on samples from 30 subjects with acute open fracture wounds (Hannigan et al., 2014). The most abundant genera observed are, in descending order, *Staphylococcus, Corynebacterium, Streptococcus, Acinetobacter, Anaerococcus*, and *Pseudomonas*. This study reports that the microbiome of acute wounds becomes more similar to the peri-wound microbiome with time and healing progression (Hannigan et al., 2014).

A 2013 study by Fromantin used culture-based techniques to find that the most common genera in MFW, in no particular order are: *Staphylococcus, Pseudomonas, Corynebacterium, Streptococcus, Proteus, Escherichia, Enterococcus*, and others. This study also identified several anaerobic strains in MFWs, none of which with any prevalence greater than the others (Fromantin et al., 2013).

All four above studies report *Staphylococcus, Streptococcus*, and *Corynebacterium* as being prevalent genera in various wounds, which may make identifying target treatments for infected cutaneous wounds easier. However, comparing the results of the Fromantin study on MFW with the others may not yield generalizable conclusions because of the difference in data collection: culture-based vs. 16S rRNA gene sequencing. This discrepancy in data collection highlights the importance of new studies using 16S rRNA gene sequencing to evaluate the microbiome of MFWs.

Biofilms in MFWs have been observed to contribute to the overall burden the wounds bear on the patient, but limited methods exist to treat for biofilms. A 2009 study using a murine wound model found that biofilms composed of *Staphylococcus* significantly slowed reepithelialization compared to a control, uninfected wound (Schierle et al., 2009). The authors hypothesize that in chronic wounds, biofilms delay reepithelialization through physical barrier and inducing chronic inflammation. Biofilm presence has been said to disrupt healing because of the consequent continuous activation of the innate immune system, which further delays the proliferative phase of healing (Johnson et al., 2018). Fromantin et al. (2013) found that all four of the patients that exhibited partial reepithelialization lacked the presence of a biofilm, which also points toward the negative effect biofilm has on wound healing (Fromantin et al., 2013). To combat the effects of the biofilm, some care teams have used antimicrobial products, like cleansing solutions or antimicrobial dressings, in an attempt to disturb the biofilm and ameliorate the conditions for reepithelialization (Fromantin et al., 2014) but biofilms appear to be resistant to antibiotics. The Schierle et al. (2009) study found that biofilm-infected wounds were resistant to topical antibiotics, but not to quorum sensing inhibitors like RIP. When RIP was applied to the biofilm-infected wounds, uninfected wound healing rates seemed to be restored and bacterial bioburden in the wound was significantly decreased (Schierle et al., 2009). This implies that current methods of applying a wide variety of topical antibiotics to even acute wounds are not effective in disturbing biofilm.

### Role of Bacterial Metabolites

The odor that accompanies MFWs has been associated with the presence of dimethyl trisulfide (DMTS), a compound known to be produced by some microorganisms, and four fatty acid volatiles (Shirasu et al., 2009). DMTS presence in MFWs is not associated with a particular strain of bacteria (Shirasu et al., 2009). The four fatty acid volatiles (acetic acid, isobutyric acid, butyric acid, and isovaleric acid) are associated with different components of the MFW odor: sour, cheese, cheese, and vomit, and cheese and foot, respectively (Shirasu et al., 2009). These fatty acid volatiles are associated with the anaerobic bacteria that thrive in necrotic tissue, often found in MFWs (Shirasu et al., 2009). Pungent odors are produced when these fatty acids combine with amines and diamines like cadaverine and putrescine that are byproducts of the proteolytic bacteria present (Gethin, 2011). The precise etiology of MFW malodor has not been identified.

Bacterial metabolites in MFW exudate have been thought to contribute to the degradation of the periwound skin (Tamai et al., 2013, 2016). Tamai et al. (2013) reported that 58.3% of 24 patients studied with MFWs and primary breast cancer had exudate-related moisture-associated dermatitis (MAD) (Tamai et al., 2013). Results of the 2016 study by Tamai et al. detailed components of exudate that are associated with MAD. The one statistically significant difference between variables studied in the non-MAD and MAD group was the presence of the polyamines putrescine (PUT) and cadaverine (CAD) (Tamai et al., 2016). These authors found significantly higher levels of PUT in the MAD group than the non-MAD and only detected CAD in the MAD group. Polyamines are synthesized by some bacteria, including *E. coli*, and are associated with skin irritation in high doses (Tamai et al., 2016). PUT is a polyamine with many functions, including promoting cell proliferation and DNA synthesis. Tamai et al. (2016) hypothesized that the higher levels of PUT in MAD patients was due to the higher activity of cell proliferation in the MFWs of these patients. These authors hypothesized that the thick necrotic tissue present in MAD patients and associated elevated levels of anaerobic bacteria result in higher levels of CAD (Tamai et al., 2016).

## Discussion

**Figure 2:**
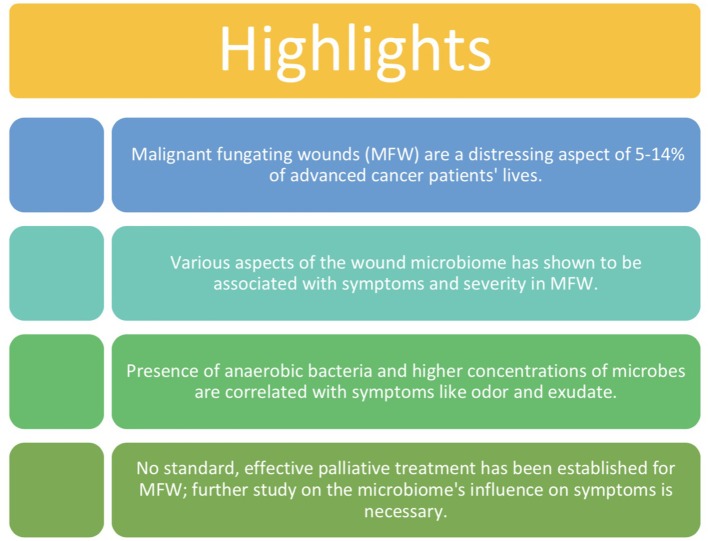
Key points of this literature review.

The infrequency of MFWs relative to other chronic wounds, limited studies by small sample size and lack of an untreated control group (Lund-Nielsen et al., 2011) has historically made them difficult to study in depth (Figure 2). A large, collaborative project with the participation of several institutions and use of 16S rRNA gene sequencing is needed to yield generalizable and useful results regarding the most effective method of treatment (Fromantin et al., 2014). Significant gaps in our understanding of the relationship between the MFW microbiome and symptoms and their severity necessitate further extensive study. These gaps include a lack of identification of specific microbes as symptom etiology and a lack of understanding of host immune response to infection and colonization in MFWs. The current studies utilize culture-based techniques to characterize the microbiome of MFWs, which is an inadequate method to describe the complexity of microbiomes (Misic et al., 2014). Instead, 16S rRNA gene sequencing, metagenomics, and metabolomics will be used by the proposed study to identify specific microbes and their metabolites of the cutaneous wound microbiome as potential therapeutic targets. This will allow for a comprehensive description of polymicrobial life in MFWs, which is essential to identify specific targets for developing a treatment.

## Author Contributions

MV wrote the bulk of the review and contributed to editing. ZF, CT, MF, CC, and SS contributed to editing of the review. XL and DS came up with the design and concept for the review and contributed to writing and editing.

### Conflict of Interest

The authors declare that the research was conducted in the absence of any commercial or financial relationships that could be construed as a potential conflict of interest.
